# Efficacy and safety of direct oral anticoagulants in patients with venous thrombosis and inherited thrombophilia

**DOI:** 10.7150/ijms.108258

**Published:** 2025-06-23

**Authors:** Amir Warwar, Iren Zargari, Nili Stein, Ibrahim Zoubi, Emad Muhammad, Shoshan Perek, Marwa Naamneh, Meir Preis, Walid Saliba

**Affiliations:** 1 Institute of Hematology, Lady Davis Carmel Medical Center, Haifa, Israel.; 2 Ruth and Bruce Rappaport Faculty of Medicine, Technion-Israel Institute of Technology, Haifa, Israel.; 3 Department of Internal Medicine B, Lady Davis Carmel Medical Center, Haifa, Israel.; 4 Department of Community Medicine and Epidemiology, Lady Davis Carmel Medical Center, Haifa, Israel.

**Keywords:** Inherited thrombophilia, venous thromboembolism, venous thrombosis, direct oral anticoagulants

## Abstract

**Introduction:** Inherited thrombophilia screening is widely performed in patients with venous thromboembolism (VTE). Although recent studies suggest that direct oral anticoagulants (DOACs) may provide comparable efficacy and safety to Vitamin K antagonists (VKAs) in this population, robust evidence to support their extensive use is still lacking. We aimed to evaluate the rates of VTE recurrence and overall bleeding in patients with inherited thrombophilia treated with DOACs versus VKAs, with particular interest in those with severe thrombophilia.

**Methods:** Using the electronic database of the largest healthcare provider in Israel, we conducted a retrospective search for patients with a recorded VTE between 2012 and 2021 (the index event). Patients aged 18 or older at the time of diagnosis were included if they began treatment with either a DOAC or a VKA within 30 days of the index event, provided they had laboratory evidence of inherited thrombophilia. Patients were followed up for two independent outcomes (VTE recurrence and overall bleeding) until December 31, 2022 or until termination of follow-up due to death, switching from one oral anticoagulation class to another, or discontinuation of oral anticoagulation. Rates of VTE recurrence and overall bleeding were compared using Cox regression and reported as hazard ratios (HRs) with 95% confidence intervals (CIs).

**Results:** A total of 398 patients (median age 50.9±17.8, males 51.8%, severe thrombophilia 24.9%) were included. Among these, 230 patients (57.8%) were prescribed DOACs, while 168 patients (42.2%) received VKAs. The median follow-up for VTE recurrence and overall bleeding was 21.1 months and 20 months, respectively. Using the VKAs group as a reference, the hazard ratio for VTE recurrence on DOACs was 1.25 (95% CI, 0.23-6.7), and the hazard ratio for overall bleeding on DOACs was 0.33 (95% CI, 0.03-3.7). Restricting the analysis to 99 patients with severe thrombophilia (46 on DOACs, 53 on VKAs) showed no substantial differences in both efficacy and safety.

**Conclusions:** Among patients with inherited thrombophilia treated with DOACs or VKAs, this study found no significant difference in the risk of recurrent VTE and observed a non-significant trend toward a lower risk of bleeding with DOACs.

## Introduction

Venous thromboembolism (VTE) commonly manifests as deep vein thrombosis (DVT) and/or pulmonary embolism (PE) at an annual incidence of about 1 per 1000 adults. The tendency to develop VTE rises rapidly after the age of 45, and reaches an incidence rate of 5-6 per 1000 by the age of 80^1^.

Inherited thrombophilia is a genetically determined predisposition to develop VTE^2^, which may lead to a higher incidence of thrombosis, including in atypical sites and at a younger age^3^. In most cases, inherited thrombophilia screening pertains to five known defects in the coagulation system: Factor V Leiden (FVL), Prothrombin G20210A mutation (PGM), and deficiencies of protein S (PS), protein C (PC) and antithrombin (AT)^4^. While it is acknowledged that many other genetic causes affect the risk of thrombosis, other potential culprits have not been studied thoroughly, possibly due to their rarity^5^.

Commonly, inherited thrombophilia is classified as either mild or severe. Mild thrombophilia refers to heterozygous FVL or PGM, while severe thrombophilia encompasses homozygous FVL or PGM, compound heterozygosity for both FVL and PGM, as well as deficiencies in PS, PC or AT, along with combined defects^6^. A recent systemic review and meta-analysis by Alnor et al. found that the overall risk of first and recurrent VTE in patients with PS, PC and AT deficiencies is lower than previously reported, posing only an intermediate risk, with significant variations among patients, likely due to the varying thrombogenicity of the underlying genetic mutations^7^.

Testing for inherited thrombophilia is widely performed almost routinely^8^. However, guidelines recommend testing only in very specific clinical scenarios and thus the majority of patients with VTE should not be tested for thrombophilia^8^-^11^, as it rarely influences treatment decisions^12^. This is partly because the risk of recurrent VTE in patients with any inherited thrombophilia, compared to those without, is relatively low (RR, 1.56; 95% CI, 1.31-1.86)^11^.

In the past few years, direct oral anticoagulants (DOACs), which directly inhibit either thrombin (dabigatran) or factor Xa (rivaroxaban, apixaban, and edoxaban), have been introduced into clinical practice for the prevention and treatment of VTE. Large phase III clinical trials have established that DOACs offer comparable or better efficacy and safety outcomes to Vitamin K antagonists (VKAs) in the treatment of patients with VTE^13^-^20^. As a result, DOACs are now preferred over VKAs in most patients with VTE, based on international guidelines^9^,^21^-^23^.

DOACs have several advantages, including fixed dosing with no need for monitoring, rapid and reliable onset of action, limited drug and food interactions, convenience for use, a short half-life, and a wide therapeutic window^24^,^25^. However, there is no robust evidence on the use of DOACs in inherited thrombophilia^26^, especially in severe forms, which are poorly represented in clinical studies^27^. A few studies investigating the efficacy and safety of DOACs in patients with VTE and inherited thrombophilia reported no major differences in comparison to VKAs. However, many of these studies have limitations that may hinder their generalizability, including underrepresentation of apixaban^26^,^28^-^33^, lack of information on FVL and PGM zygosity status^26^,^32^-^35^, and co-inclusion of a heterogenous patient population with conditions that typically are not tested for inherited thrombophilia, such as antiphospholipid syndrome^26^,^32^-^34^ and superficial vein thrombosis^34^,^35^.

This retrospective cohort study aimed to provide real-world data on the rates of VTE recurrence and overall bleeding in patients with inherited thrombophilia treated with DOACs compared to those treated with VKAs, with particular interest in patients with severe thrombophilia and those treated with apixaban, as it is the most commonly used DOAC in Israel^36^.

## Methods

### Data acquisition

Data for this study were acquired from the computerized database of Clalit Health Services (CHS), the largest healthcare provider in Israel. The electronic medical record database of CHS includes data from multiple sources, such as records from primary care physicians, specialty clinics, hospitals, laboratories and pharmacies. Diagnoses are captured in the chronic disease registry of CHS by diagnosis-specific algorithms that incorporate International Classification of Diseases Nineth revision (ICD-9) coding, text reading, laboratory test results, and the registration of all prescription medications, whether dispensed by physicians or acquired by patients.

### Study design and population

Using the CHS database, we conducted a retrospective search for patients with a recorded VTE between January 1, 2012, and December 31, 2021 (the index event). Patients aged 18 or older at the time of diagnosis were included if they began treatment with either a DOAC or a VKA within 30 days of the index event, provided they had laboratory evidence of inherited thrombophilia. Patients who had used any type of anticoagulant in the 90 days pritaor to the index event were excluded, as were those with antiphospholipid syndrome, liver cirrhosis, or a glomerular filtration rate (GFR) of less than 30 mL/min.

Patients were divided into two groups, based on whether they received a DOAC or a VKA, and were retrospectively followed until December 31, 2022, or until earlier termination of follow-up due to death, switching from one oral anticoagulation class to another (DOAC to VKA, or vice versa), or discontinuation of the initial oral anticoagulant (OAC), defined as lack of prescription retrieval within 30 days from the date of the last supplied pill. This 30-day grace period was primarily granted to accommodate temporary discontinuations of OACs due to medical circumstances, such as hospitalization (where medications are typically be provided by the hospital) and invasive procedures (which may necessitate a temporary interruption). Notably, during follow-up, transitioning to a different type of OAC from the same class (for example, switching from apixaban to rivaroxaban) was permitted. Temporary use of heparin, low molecular weight heparin (LMWH) or fondaparinux was also allowed, as long as the initial OAC was obtained within 30 days of the previous prescription. This study was approved by a centralized institutional review board committee.

### Variables and outcome definitions

For each patient included in the study, baseline data were collected from the CHS database, including demographic variables, risk factors, selected chronic medical conditions, and prescription medications, as well as data regarding the type of anticoagulant used, inherited thrombophilia workup, and the index VTE event. Data on prescriptions of OACs were gathered to assess continued follow-up eligibility. Patients were followed for two independent outcomes: recurrent VTE (efficacy endpoint), defined as DVT, PE, and atypical-site thrombosis (detailed in **Table S1**); and overall bleeding (safety endpoint), defined as intracranial hemorrhage, gastrointestinal bleeding, and bleeding from other sites (elaborated in **Table S2**).

### Inherited thrombophilia diagnosis

Inherited thrombophilia testing was sought for in every patient with an index event recorded between 2012 and 2021. Since inherited thrombophilia is congenital, we assumed that a positive result can be drawn from any period of the patient's life. Therefore, medical records were scanned for thrombophilia testing from the availability of the Clalit database in 2003, until the end of follow-up in 2022. Polymorphisms of FVL and PGM were considered positive if reported as either heterozygous or homozygous. Deficiencies of natural anticoagulants (i.e., PS, PC, and AT) were deemed positive if activity / antigen levels were below 60% on at least two occasions, with no levels of 60% or higher ever recorded. Additional data from the medical records of all patients who tested positive for deficiency of natural anticoagulants were collected to extract information regarding the circumstances under which the tests were conducted. Results for PS, PC and AT levels obtained while on anticoagulation, in an acute setting, or in proximity to a thrombotic event, were disregarded. Specifically for PS results in women, all tests drawn during pregnancy or postpartum, as well as those obtained while using hormonal contraceptives, were also ignored, due to the possibility of false positives^10^.

### Statistical methods

Continuous variables were summarized with mean ± standard deviation, and categorical variables were presented as numbers and proportions. Comparisons of baseline characteristics between DOACs and VKAs were performed using the Chi-square test for categorical variables and independent t-test for continuous variables. The annualized incidence rates of VTE recurrence and overall bleeding were estimated by dividing the number of incident cases of these outcomes by the total follow-up time and were expressed as number per 1000 person-years. Cox proportional hazard regression models were used to assess the association between medication type and the study outcomes and to estimate the crude hazard ratios (HRs) along with 95% confidence interval (95% CI). P < 0.05 was considered to be statistically significant. Statistical analyses were conducted using IBM SPSS Statistics version 28.0 (IBM, Armonk, NY, USA).

## Results

### Patients' characteristics

**Figure 1:**
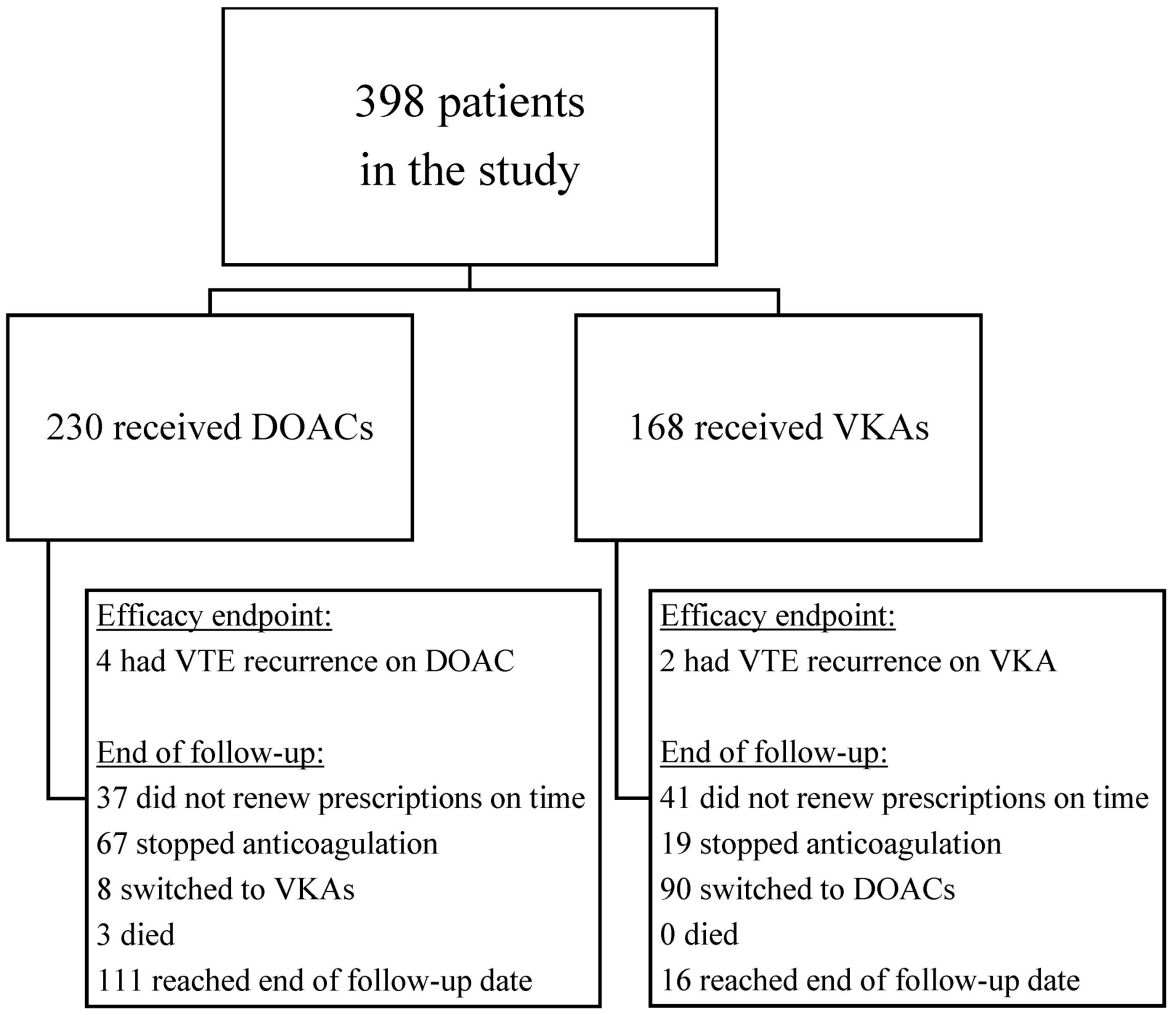
Flowchart of study population and reasons for end of follow-up (for efficacy endpoint)

After implementing the exclusion criteria and careful reviewing of thrombophilia results, we identified 398 patients with inherited thrombophilia who experienced a VTE event between 2012 and 2021 and were started on oral anticoagulation within 30 days of the index event. Among these, 230 patients (57.8%) were prescribed DOACs, while 168 patients (42.2%) received VKAs (**Figure 1**). The demographic characteristics and clinical risk factors for VTE recurrence and bleeding are presented in **Table 1**. In this study, the median age of patients was 50.9±17.8 years, with a significant difference between the two groups: patients on VKAs were generally younger (47.3±17.8 years) than those on DOACs (53.5±17.4 years). Males and females accounted for 51.8% and 48.2% of the study population, respectively. In comparison to the VKAs group, people prescribed DOACs were more likely to have typical cardiovascular risk factors, including obesity (36.1% vs. 24.4%), hypertension (30% vs. 27.4%), hyperlipidemia (47.4% vs. 42.9%) and diabetes mellitus (15.7% vs. 12.5%). The VKAs group had a higher prevalence of both baseline venous and arterial thrombosis, including prior VTE (11.3% vs. 6.5%), stroke or transient ischemic attack (TIA) (8.3% vs. 3.5%) and ischemic heart disease (8.3% vs. 7.8%). The rates of antiplatelet drug use prior to the index event did not differ significantly between the two groups. A diagnosis of malignancy was more prevalent in the DOACs group (12.2%), compared to the VKAs group (6%).

As shown in **Table 2**, DVT was the most common site of VTE (63.1%), followed by PE (27.1%). About three-quarters of the study participants had mild thrombophilia, while approximately one-quarter had severe thrombophilia. The proportion of patients with severe thrombophilia was higher in the VKAs group (31.5% vs. 20%). The most commonly prescribed DOAC after the index event was apixaban (53.5%), followed by rivaroxaban (44.8%). All patients treated with VKAs received Warfarin.

### Efficacy outcomes

**Figure 2:**
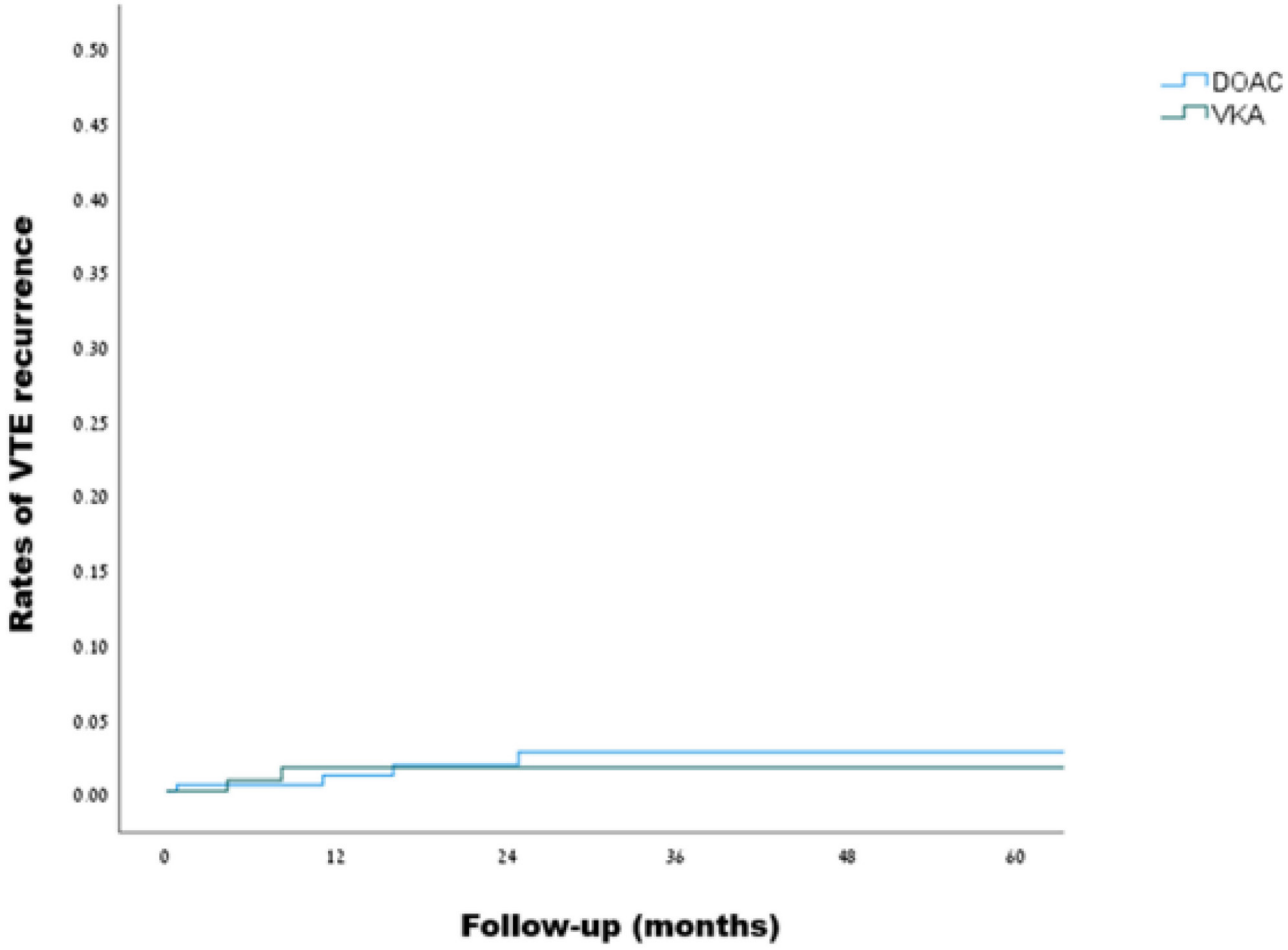
Kaplan-Meier curves for VTE recurrence in patients with inherited thrombophilia (DOACs vs. VKAs, log rank: p=0.795)

Over a median follow-up of 21.1 months, VTE recurrence was observed in 4 out of 230 patients in the DOACs group during 558 person-years of follow-up, and in 2 out of 168 patients in the VKAs group during 448 person-years of follow-up, as detailed in **Table 3**, reflecting a crude incidence rate for recurrent VTE of 7.2 (95% CI, 2.3-17.3) and 4.5 (95% CI, 0.7-14.8) per 1000 person-years, respectively. Using the VKAs group as a reference, the hazard ratio for VTE recurrence on DOACs was 1.25 (95% CI, 0.23-6.7, p=0.796). Kaplan-Meir curves illustrating the rates of VTE recurrence are presented in **Figure 2**. Investigation of the two cases of VTE recurrence in the VKAs group revealed subtherapeutic international normalized ratios (INRs) at the time of events in both cases. Of the 4 VTE recurrences in the DOACs group, one occurred with rivaroxaban and three with apixaban. Notably, two of the events involved low-dose anticoagulation - one of which in a patient with FVL homozygosity (more details are given in **Table S3**). A subgroup analysis of patients with severe thrombophilia (99 patients overall), revealed one VTE recurrence in 46 patients in the DOACs group during a follow-up of 103 person-years, and one VTE recurrence in 53 patients in the VKAs group during a follow-up of 138 person-years, with a hazard ratio of 0.98 (95% CI, 0.06-15.6, p = 0.987).

### Safety outcomes

**Figure 3:**
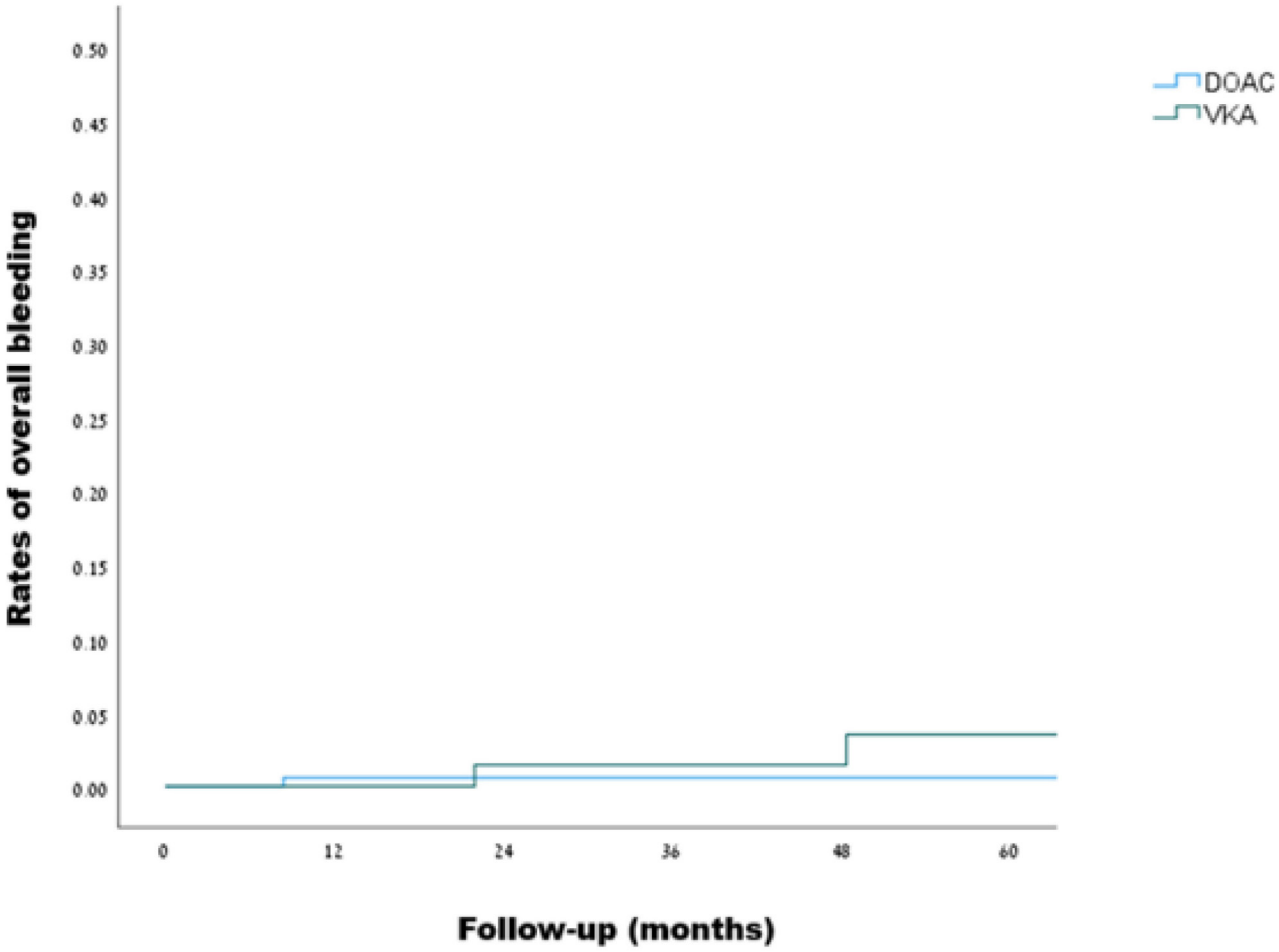
Kaplan-Meier curves for overall bleeding events in patients with inherited thrombophilia (DOACs vs. VKAs, log rank: p=0.348)

Over a median follow-up of 20 months, overall bleeding was observed in 1 out of 230 patients in the DOACs group during 561 person-years of follow-up, and in 2 out of 168 patients in the VKAs group during 448 person-years of follow-up, as detailed in **Table 4**, reflecting a crude incidence rate for overall bleeding of 1.8 (95% CI, 0.09-8.8) and 4.5 (95% CI, 0.7-14.8) per 1000 person-years, respectively. Using the VKAs group as a reference, the hazard ratio for overall bleeding on DOACs was 0.33 (95% CI, 0.03-3.7, p = 0.372). Kaplan-Meir curves demonstrating the rates of overall bleeding are presented in **Figure 3**. Investigation of the bleeding episodes revealed two episodes of major intracranial hemorrhage (subdural and subarachnoid) in the VKAs group, one of which occurred following a motor vehicle accident, and one episode of non-major gastrointestinal bleeding in the DOACs group, in a patient receiving apixaban. Two out of the three outcomes occurred in patients with mild thrombophilia, while one occurred in a patient with severe thrombophilia (more details can be found in **Table S4**).

## Discussion

This study shows no significant difference in the risk of recurrent VTE between patients with inherited thrombophilia treated with DOACs or VKAs, along with a non-significant trend toward a lower risk of bleeding with DOACs. An intriguing observation from this study is that since the introduction of DOACs, most patients with inherited thrombophilia have been either prescribed DOACs or transitioned to them. Our study demonstrated that, over a follow-up period 21.1 months, the vast majority of patients with inherited thrombophilia treated with DOACs rarely experienced breakthrough thrombosis, especially when prescribed full-dose anticoagulation and adhered to treatment.

Our study results are in line with the findings of a meta-analysis published in 2017 by Elsebaie et al., who examined 1994 inherited thrombophilia patients with VTE from eight randomized controlled trials comparing DOACs to VKAs. They reported a non-significant trend toward lower rates of both VTE recurrence (RR 0.70, 95% CI 0.34-1.44) and all bleeding events (RR 0.92, 95% CI 0.62-1.36) in patients on DOACs compared to those on VKAs^26^. Comparable results in terms of efficacy were observed in a prospective study by Campello et al., with a HR of 0.67 (95% CI 0.16-2.77) for VTE recurrence in patients treated with DOACs versus those on VKAs or heparins. However, unlike our study, overall bleeding was significantly increased in the DOACs group (HR 2.24, 95% CI 1.10-4.58), while major bleeding events were observed only in the VKAs group^28^.

In this study, we defined VTE as DVT, PE and atypical-site thrombosis. Unlike other studies^34^,^35^, we purposefully excluded patients with superficial vein thrombosis, as this condition is more often associated with local rather than systemic thrombotic risk factors and typically does not warrant testing for thrombophilia^37^. We also excluded all patients diagnosed with antiphospholipid syndrome, as including this subset would significantly confound the results of a study focused on the inherited thrombophilia population. With these adjustments, we believe that our study population accurately represents the typical VTE patient tested for inherited thrombophilia.

One of the primary objectives of this study was to evaluate the efficacy and safety of DOACs specifically in patients with severe thrombophilia. We found that in many studies, this group of patients is often indistinctly combined with those having mild thrombophilia, where, for instance, all FVL variants would be included in the same analysis, regardless of zygosity^26^,^32^-^35^. Many case reports and case series have observed favorable efficacy of DOACs in patients with PS, PC, and AT deficiency associated with venous thrombosis, including atypical-site thrombosis^38^-^47^. However, some reports have documented DOAC failure in this high-risk subgroup, in some cases despite full-dose anticoagulation and consistent adherence^45^-^51^. High-risk variants of FVL and PGM were investigated by Dan et al., who followed 56 homozygous and compound heterozygous carriers of FVL and PGM treated with either a DOAC or Warfarin. Their findings, although not statistically significant, showed a low rate of VTE recurrence in both groups, suggesting that DOACs may be an effective option in this clinical setting^52^. A recent review by Kovac et al. explored the data available on severe thrombophilia patients and concluded that, in general, full-dose DOACs have effects comparable to VKAs. However, they observed higher rates of treatment failure with DOACs in patients with severe PS deficiency (below 20%) and homozygous AT deficiency Budapest 3, warranting caution in these populations^53^. In our study, about a quarter of the patients had severe thrombophilia, and although this study was not powered to detect significant differences between DOACs and VKAs in this population, we did not observe major differences in efficacy and safety between the two groups.

Another important goal of the study was to gather data on apixaban in patients with inherited thrombophilia, as it is the most commonly used DOAC in Israel^36^, and is considered the most cost-effective anticoagulant, according to the 2020 NICE Venous Thromboembolism guideline^9^. In various studies, apixaban has either been omitted^26^,^32^,^33^ or insufficiently included, with its representation ranging from 0% to 25% in most cohorts and case series^28^-^30^. In our study, more than 50% of DOAC users were prescribed apixaban, which leads us to believe that the study results may be applicable to our daily practice.

Compared to other studies^26^,^30^,^32^-^35^,^52^, we applied stricter exclusion criteria, omitting patients with antiphospholipid syndrome, superficial venous thrombosis, and arterial thrombosis to focus on the typical VTE patient. Additionally, we clearly distinguished between mild and severe thrombophilia, allowing for a more precise evaluation of efficacy and safety outcomes in each group.

The low incidence rates of VTE recurrence and overall bleeding observed in our study provide reassuring results regarding both the safety and efficacy of DOACs, particularly apixaban and rivaroxaban, in patients with thrombophilia, including those with severe thrombophilia. Our analysis yielded wide CIs, reflecting the rarity of events and the small sample size in the study. Although statistical significance was not reached, an appreciable 67% reduction in overall bleeding risk (HR=0.33) was observed in the DOACs group compared to the VKAs group. Regarding VTE recurrence, the difference between the two groups was not statistically significant, and the magnitude of association appears modest. Larger studies are needed to confirm these safety-related observations and to establish conclusive evidence regarding the effectiveness of DOACs. Overall, our findings suggest that DOACs may be a viable therapeutic option for both mild and severe thrombophilia and could serve as an alternative to VKAs in clinical practice.

This study has a few limitations: we used an administrative database that was not specifically created for this research. Although thrombophilia results and study outcomes were manually validated, extracting diagnoses using the ICD-9 coding system is not flawless and may introduce inaccuracies in some study parameters and results. Additionally, diagnoses of natural anticoagulant deficiencies were based on laboratory measurements rather than genetic testing, which was unavailable. This may have partially contributed to false-positive diagnoses of PS, PC, and AT deficiencies. However, these limitations are unlikely to have disproportionately affected one group over the other. Another limitation is that data on changes in doses of DOACs over time and the time in therapeutic range (TTR) for Warfarin were not collected. In addition, compliance with DOACs and VKAs was not assessed directly through a review of medical follow-ups but rather indirectly by tracking the timely retrieval of prescriptions with a 30-day grace period, which may have resulted in imprecise adherence rates. Finally, we defined overall bleeding as a safety endpoint by incorporating clinical outcomes, such as intracranial hemorrhage, gastrointestinal bleeding and other bleeding sites. However, due to the study design, we did not assess changes in hemoglobin levels or the administration of blood products, preventing us from utilizing the ISTH criteria for either major bleeding^54^ or clinically relevant non-major bleeding (CRNMB)^55^.

## Conclusions

Among patients with inherited thrombophilia treated with DOACs or VKAs, this study found no significant difference in the risk of recurrent VTE and observed a non-significant trend toward a lower risk of bleeding with DOACs.

## Supplementary Material

Supplementary tables.

## Figures and Tables

**Table 1:** Baseline demographic and clinical characteristics of patients in the study

Variable	Total (n=398)	DOACs (n=230)	VKAs (n=168)	p-value
Age	50.9±17.8	53.5±17.4	47.3±17.8	< 0.001
Male sex	206 (51.8)	121 (52.6)	85 (50.6)	0.691
Socioeconomical status:				0.908
- Low	138 (24.7)	78 (33.9)	60 (35.7)	
- Middle	157 (39.4)	89 (38.7)	68 (40.5)	
- High	98 (24.6)	60 (26.1)	38 (22.6)	
- Missing	5 (1.3)	3 (1.3)	2 (1.2)	
Smoking	156 (39.2)	90 (39.1)	66 (39.3)	0.975
Obesity	124 (31.2)	83 (36.1)	41 (24.4)	0.013
Hypertension	115 (28.9)	69 (30.0)	46 (27.4)	0.569
Hyperlipidemia	181 (45.5)	109 (47.4)	72 (42.9)	0.370
Diabetes Mellitus	57 (14.3)	36 (15.7)	21 (12.5)	0.375
Heart failure	3 (0.8)	2 (0.9)	1 (0.6)	> 0.99
Atrial Fibrillation	12 (3.0)	8 (3.5)	4 (2.4)	0.527
Ischemic Heart Disease	32 (8.0)	18 (7.8)	14 (8.3)	0.854
Stroke / TIA*	22 (5.5)	8 (3.5)	14 (8.3)	0.036
Malignancy	38 (9.5)	28 (12.2)	10 (6.0)	0.037
IBD✝	3 (0.8)	1 (0.4)	2 (1.2)	0.576
Renal failure (GFR:30-59 mL/min)	18 (4.5)	10 (4.3)	8 (4.8)	0.844
Prior VTE	34 (8.5)	15 (6.5)	19 (11.3)	0.091
Aspirin use	66 (16.6)	38 (16.5)	28 (16.7)	0.969
P2Y12 Inhibitor use	13 (3.3)	8 (3.5)	5 (3.0)	0.781

* TIA - Transient Ischemic Attack✝ IBD - Inflammatory Bowel Disease

**Table 2:** Baseline data of index VTE, thrombophilia and OACs in the study

Variable	Total (n=398)	DOACs (n=230)	VKAs (n=168)	p-value
**Index VTE site**				0.27
- DVT	251 (63.1)	148 (64.3)	103 (61.3)	
- PE	108 (27.1)	58 (25.2)	50 (29.8)	
- Atypical site VTE	15 (3.8)	8 (3.4)	7 (4.3)	
- ≥ 2 sites	24 (6.0)	16 (7.0)	8 (4.8)	
**Thrombophilia**				0.08
**Mild thrombophilia**	299 (75.1)	184 (80)	115 (68.4)	
- FVL Heterozygous	211	130	81	
- PGM Heterozygous	88	54	34	
**Severe thrombophilia**	99 (24.9)	46 (20)	53 (31.5)	
- FVL Homozygous	23	9	14	
- PGM Homozygous	4	2	2	
- Compound FVL + PGM Heterozygous	21	10	11	
- Protein S deficiency	33	18	15	
- Protein C deficiency	10	4	6	
- Antithrombin deficiency	2	0	2	
- Combined	4	3	1	
**Mean levels of natural anticoagulants**				
- Protein S antigen	N=3735% +/- 12.7%	N=2136.9% (12.3%-53.8%)	N=1632.5% (11.7%-47.7%)	0.206
- Protein C activity	N=1045.4% +/- 7.3%	N=449.5% (48.5-51%)	N=642.6% (29.5-52%)	0.257
- Antithrombin	N=237.7% +/- 4.6%	N=0	N=237.7% (32-45%)	
**Oral anticoagulant**				
- Apixaban		123 (53.5%)		
- Rivaroxaban		103 (44.8%)		
- Dabigatran		4 (1.7%)		
- Warfarin			168 (100%)	

**Table 3:** VTE recurrence in patients with inherited thrombophilia (DOACs vs. VKAs):

OAC	Number	Events	Follow-up (person-years)	Incidence per 1000 person years	HR (95% CI)	p-value
DOACs	230	4	558	7.2 (2.3-17.3)	1.25 (0.23-6.7)	0.796
VKAs	168	2	448	4.5 (0.7-14.8)	Reference	

**Table 4:** Overall bleeding events in patients with inherited thrombophilia (DOACs vs. VKAs):

OAC	Number	Events	Follow-up (person-years)	Incidence per 1000 person years	HR (95% CI)	p-value
DOACs	230	1	561	1.8 (0.09-8.8)	0.33 (0.03-3.7)	0.372
VKAs	168	2	448	4.5 (0.7-14.8)	Ref	

## Abbreviations

VTE: Venous thromboembolism
DOACs: Direct oral anticoagulants
VKAs: Vitamin K antagonists
DVT: Deep vein thrombosis
PE: Pulmonary embolism
FVL: Factor V Leiden
PGM: Prothrombin Gene Mutation G20210A
PS: Protein S
PC: Protein C
AT: Antithrombin
CHS: Clalit Health Services
ICD-9: International Classification of Diseases Nineth revision
GFR: Glomerular filtration rate
OAC: Oral anticoagulant
LMWH: Low molecular weight heparin
TIA: Transient ischemic attack
INR: International normalized ratio
IBD: Inflammatory bowel disease
TTR: Time in therapeutic range
ISTH: International Society on Thrombosis and Haemostasis
CRNMB: Clinically relevant non-major bleeding
