# Supplementary material for: Efficacy and safety of direct oral anticoagulants in patients with venous thrombosis and inherited thrombophilia
Source: Int J Med Sci. 2025 Jun 23;22(13):3182–90. doi: 10.7150/ijms.108258 (PMC12320646; doi:10.7150/ijms.108258)
Supplement: Supplementary file 1 — Supplementary tables. [file ijmsv22p3182s1.pdf]

Table S1:

ICD-9 codes for diagnosis of "venous thromboembolism" (VTE)

**Deep vein thrombosis:**	
451.1	Phlebitis and thrombophlebitis of deep veins of lower extremities
451.11	Phlebitis and thrombophlebitis of unspecified femoral vein
451.19	Phlebitis and thrombophlebitis of deep veins of lower extremities, other
451.81	Phlebitis and thrombophlebitis of iliac vein
451.83	Phlebitis and thrombophlebitis of deep veins of upper extremities.
453.4	Venous embolism and thrombosis of deep vessels of lower extremity
453.40	Acute venous embolism and thrombosis of unspecified deep vessels of lower extremity
453.41	Acute venous embolism and thrombosis of deep vessels of proximal lower extremity
453.42	Acute venous embolism and thrombosis of deep vessels of distal lower extremity
453.82	Acute venous embolism and thrombosis of deep veins of upper extremity
453.84	Acute venous embolism and thrombosis of axillary veins
453.85	Acute embolism and thrombosis of unspecified subclavian vein
**Pulmonary embolism:**	
415.1	Pulmonary embolism and infarction
415.11	Iatrogenic pulmonary embolism & infarction
415.13	Saddle embolus of pulmonary artery
415.19	Other pulmonary embolism & infarction
**Cerebral vein thrombosis:**	
325	Phlebitis and thrombophlebitis of intracranial venous sinuses
437.6	Nonpyogenic thrombosis of intracranial venous sinus
**Portal / Hepatic vein thrombosis:**	
452	Portal vein thrombosis
453.0	Budd-chiari syndrome
**Renal vein thrombosis:**	
453.3	Embolism and thrombosis of renal vein
**Other venous thrombosis:**	
453.2	Other venous embolism and thrombosis of inferior vena cava
453.86	Acute venous embolism and thrombosis of internal jugular veins
453.87	Acute venous embolism and thrombosis of other thoracic veins

Table S2:

ICD-9 codes for diagnosis of "overall bleeding"

**Gastrointestinal bleeding:**	
456.0	Esophageal varices with bleeding
456.20	Varices in diseases classified elsewhere with bleeding
530.82	Esophageal hemorrhage
531.0x	Gastric ulcer, acute with haemorrhage
531.2x	Gastric ulcer, acute with haemorrhage and perforation
531.4x	Gastric ulcer, chronic or unspecified with haemorrhage
531.6x	Gastric ulcer, chronic or unspecified with haemorrhage and perforation
532.0x	Duodenal ulcer, acute with haemorrhage
532.2x	Duodenal ulcer, acute with haemorrhage and perforation
532.4x	Duodenal ulcer, chronic or unspecified with haemorrhage
532.6x	Duodenal ulcer, chronic or unspecified with haemorrhage and perforation
533.0x	Peptic Ulcer, site unspecified, with haemorrhage
533.2x	Peptic Ulcer, site unspecified, acute with haemorrhage and perforation
533.4x	Peptic Ulcer, site unspecified, chronic or unspecified with haemorrhage
533.6x	Peptic Ulcer, site unspecified, chronic or unspecified with haemorrhage and perforation
534.0x	Gastrojejunal ulcer, acute with haemorrhage
534.2x	Gastrojejunal ulcer, acute with haemorrhage and perforation
534.4x	Gastrojejunal ulcer, chronic or unspecified with haemorrhage
534.6x	Gastrojejunal ulcer, chronic or unspecified with haemorrhage and perforation
535.01	Acute gastritis, with hemorrhage
535.11	Atrophic gastritis, with hemorrhage
535.21	Gastric mucosal hypertrophy, with hemorrhage
535.31	Alcoholic gastritis, with hemorrhage
535.41	Other gastritis, with hemorrhage
535.51	Unspecified gastritis and gastroduodenitis, with hemorrhage
535.61	Duodenitis, with hemorrhage
537.83	Angiodysplasia of stomach and duodenum with hemorrhage
562.02	Diverticulosis of small intestine with hemorrhage
562.03	Diverticulitis of small intestine with hemorrhage
562.12	Diverticulosis of colon with hemorrhage
562.13	Diverticulitis of colon with hemorrhage
569.3	Hemorrhage of rectum and anus
569.85	Angiodysplasia of intestine with hemorrhage
578.x	Gastrointestinal hemorrhage
**Intracranial hemorrhage:**	
430	Subarachnoid hemorrhage
431	Intracerebral hemorrhage
432.0	Nontraumatic extradural hemorrhage
432.1	Subdural hemorrhage
432.9	Unspecified intracranial hemorrhage
852.0x	Subarachnoid hemorrhage following injury without mention of open intracranial wound

852.4x	Extradural hemorrhage following injury without mention of open intracranial wound
853.0x	Other and unspecified intracranial hemorrhage following injury
**Urogenital hemorrhage:**	
596.7x	Hemorrhage into bladder wall
599.7x	Hematuria
602.1x	Congestion and hemorrhage of prostate
620.1	Corpus luteum cyst or hematoma
621.4	Hematometra
626.2	Excessive and frequent menstruation
626.5	Ovulation bleeding
626.7	Postcoital bleeding
626.8	Other disorders of menstruation and other abnormal bleeding from female genital tract
626.9	Unspecified disorders of menstruation and other abnormal bleeding from female genital tract
**Eye-related bleeding:**	
360.43	Hemophthalmos
362.43	Hemorrhagic detachment of retinal pigment epithelium
362.81	Retinal hemorrhage
363.61	Choroidal hemorrhage, unspecified
363.62	Expulsive choroidal hemorrhage
363.72	Hemorrhagic choroidal detachment
364.41	Hyphema of iris and ciliary body
372.72	Conjunctival hemorrhage
374.81	Hemorrhage of eyelid
376.32	Orbital hemorrhage
377.42	Hemorrhage in optic nerve sheaths
379.23	Vitreous hemorrhage
**Other bleeding sites:**	
285.1	Acute posthemorrhagic anemia
423.0x	Hemopericardium
568.81	Hemoperitoneum (nontraumatic)
719.1x	Hemarthrosis
782.7	Spontaneous ecchymoses
784.7	Epistaxis
784.8	Hemorrhage from throat
786.3x	Hemoptysis

Table S3:

Patients with VTE recurrence on anticoagulation (efficacy endpoint):

**Age**	**Sex**	**Thrombophilia**	**OAC**	**VTE event**	**Comment**
55	Male	FVL Homozygous	Warfarin	Proximal DVT	Subtherapeutic INR*
26	Male	FVL Heterozygous	Warfarin	Proximal DVT	Subtherapeutic INR*
85	Female	FVL Heterozygous	DOAC (Rivaroxaban)	Proximal DVT	Full-dose DOAC
67	Female	PGM Heterozygous	DOAC (Apixaban)	Proximal DVT	Low-dose DOAC
49	Male	PGM Heterozygous	DOAC (Apixaban)	IVC Thrombus + iliofemoral DVT	Full-dose DOAC
62	Male	FVL Homozygous	DOAC (Apixaban)	Upper Limb DVT	Low-dose DOAC

*INR – International Normalized Ratio

Table S4:

Patients with bleeding episodes on anticoagulation (safety endpoints):

**Age**	**Sex**	**Thrombophilia**	**OAC**	**Bleeding event**	**Comment**
76	Male	FVL Heterozygous	Warfarin	Subdural Hemorrhage	Therapeutic INR* ; Fatal hemorrhage
38	Male	PC Deficiency	Warfarin	Subdural and Subarachnoid Hemorrhage	Therapeutic INR* ; Following motor vehicle accident
45	Male	PGM Heterozygous	DOAC (Apixaban)	Rectal Bleeding	Full-dose DOAC

*INR – International Normalized Ratio
